# Metabolomics Reveals a Role for the Chromatin-Binding Protein HMGN5 in Glutathione Metabolism

**DOI:** 10.1371/journal.pone.0084583

**Published:** 2014-01-02

**Authors:** Eric D. Ciappio, Kristopher W. Krausz, Mark Rochman, Takashi Furusawa, Jessica A. Bonzo, Lino Tessarollo, Frank J. Gonzalez, Michael Bustin

**Affiliations:** 1 Laboratory of Metabolism, National Cancer Institute, National Institutes of Health, Bethesda, Maryland, United States of America; 2 Neural Development Section, Mouse Cancer Genetics Program, National Cancer Institute, Frederick, Maryland, United States of America; University of Nebraska Medical Center, United States of America

## Abstract

High mobility group nucleosome-binding protein 5 (HMGN5) is a chromatin architectural protein that binds specifically to nucleosomes and reduces the compaction of the chromatin fiber. The protein is present in most vertebrate tissues however the physiological function of this protein is unknown. To examine the function of HMGN5 *in vivo*, mice lacking the nucleosome-binding domain of HMGN5 were generated and characterized. Serological analysis revealed that compared to wild-type littermates (*Hmgn5^+/Y^*), mice with a targeted mutation in the HMGN5 gene (*Hmgn5^tm1/Y^*), had elevated serum albumin, non-HDL cholesterol, triglycerides, and alanine transaminase, suggesting mild hepatic abnormalities. Metabolomics analysis of liver extracts and urine revealed clear differences in metabolites between *Hmgn5^tm1/Y^* and their *Hmgn5^+/Y^* littermates. *Hmgn5^tm1/Y^* mice had a significant increase in hepatic glutathione levels and decreased urinary concentrations of betaine, phenylacetylglycine, and creatine, all of which are metabolically related to the glutathione precursor glycine. Microarray and qPCR analysis revealed that expression of two genes affecting glutathione metabolism, glutathione peroxidase 6 (*Gpx6*) and hexokinase 1 (*Hk1*), was significantly decreased in *Hmgn5^tm1/Y^* mouse liver tissue. Analysis of chromatin structure by DNase I digestion revealed alterations in the chromatin structure of these genes in the livers of *Hmgn5^tm1/Y^* mice. Thus, functional loss of HMGN5 leads to changes in transcription of *Gpx6* and *Hk1* that alter glutathione metabolism.

## Introduction

High mobility group (HMGN) proteins are ubiquitously expressed in vertebrate cells and are known to affect both chromatin structure and the levels of post-translational modifications to histone tails; two important epigenetic processes involved in the regulation of gene expression [1]–[3]. The HMGN protein family contains 5 variants, named HMGN1-5, all of which bind specifically to the 147 base pair nucleosome core particle, the primary building block of chromatin, and compete among themselves and with the linker histone H1 for chromatin binding sites [4], [5]. The competitive network of interactions between HMGN proteins and histone H1 affects chromatin compaction, while the competition among HMGNs may lead to functional redundancy among individual variants [6].

Genome-wide analysis revealed that the HMGN1 variant binds preferentially to regulatory elements in the genome, such as DNase hypersensitive sites and gene promoters [7], [8] suggesting that HMGN variants can affect transcription. Indeed, several types of experiments, including analysis of genetically altered mice, revealed that either up- or down-regulation of HMGN protein variants alters the cellular transcription profile, in a variant specific and tissue specific manner [9], [10]. Conceivably, minor changes in transcription could increase the susceptibility of cells to further damage by subsequent genetic events or external stressors. For example, *Hmgn3^tm1/tm1^* mice develop glucose intolerance due to disruptions in insulin release [11], while *Hmgn1^tm1/tm1^* mice are deficient in DNA repair and also display behavioral abnormalities [12], [13]. The emerging picture suggests that while HMGN variants do not have a major impact on the transcription of specific genes or pathways, they do fine-tune the fidelity of the cellular transcription profile in a tissue- and variant- specific manner, and that loss of HMGN function can lead to detectable phenotypes. In view of these observations, it is important to examine the biological function of specific HMGN variants. Here we focus on the role of the HMGN5 variant in liver function.

HMGN5 is the most recently discovered member of the HMGN family [14], and like other HMGN variants, binds to nucleosomes, interacts with histone H1, and affects chromatin structure [15]. The gene coding for HMGN5 is located on chromosome X in both human and the mouse, and is expressed in relatively low abundance in all tissues examined [14]. HMGN5 differs from other HMGN variants in that it has a long acidic tail which enhances its ability to reduce chromatin compaction, provided that its nucleosome binding domain, located in the N-terminal region, remains intact. Disruptions of the nucleosome binding ability of the protein result in a major loss of function [14], [16]. Studies with mouse embryo fibroblasts indicated that either up- or down-regulation of HMGN5 levels leads to changes in the expression of numerous genes [9], [15].

In this study, the biological consequences of the functional loss of HMGN5 *in vivo* were determined through the use of a genetically engineered mouse that carries a targeted disruption in the nucleosome binding region of the protein. Evaluation of blood chemistries of these mice [10] suggested possible impairments in hepatic function, and metabolomic analysis of urine and liver extracts identified alterations in glutathione metabolism. Glutathione, a tripeptide molecule comprised of cysteine, glutamic acid, and glycine, is an abundant low-molecular weight thiol that plays important roles in antioxidant defense and nutrient metabolism. Gluthathione also affects the regulation of various cellular events such as cell proliferation, apoptosis, signal transduction, and immune responses [17]. Transcriptional analysis of liver tissues from *Hmgn5^+/y^* and *Hmgn5^tm1/y^* littermates revealed alterations in the expression of glutathione peroxidase 6 (*Gpx6*) and hexokinase 1 (*Hk1*), two enzymes known to be involved in glutathione metabolism [18]. This study links the expression of HMGN5 to transcriptional changes that affect glutathione metabolism in the liver.

## Experimental Proceedures

### Generation of Hmgn5^tm1/tm1^ Mice

**Figure 1:**
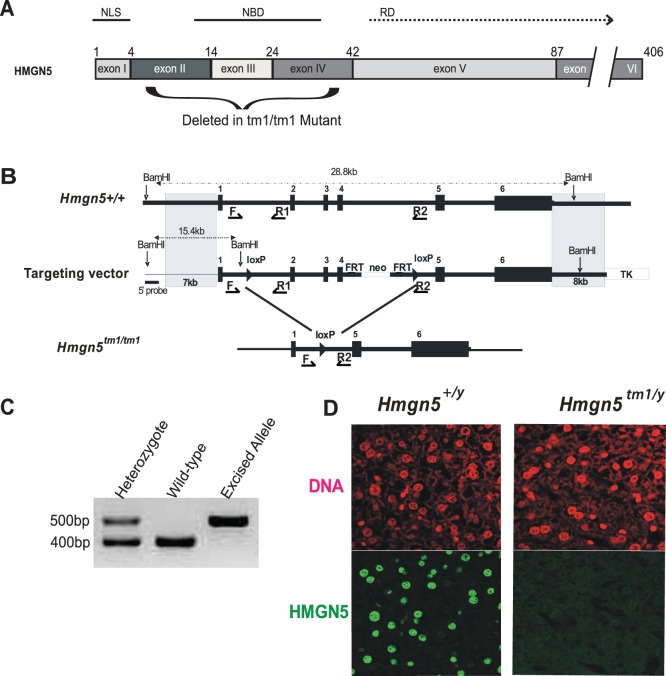
Generation of *Hmgn5^tm1/tm1^* mice. **A)** Diagram detailing the exons coding for the various functional domains of HMGN5. NLS: nuclear localization signal, NBD: nucleosome binding domain, RD: regulatory domain. The numbers denote the amino acid position at the border of each exon. The exons deleted to create the functional knock-out for HMGN5 are denoted by the curly bracket below the diagram. **B)** Strategy for generating *Hmgn5^tm1/tm1^* knockout mice. *loxP* sites were placed downstream of exon I and upstream of exon V of the *Hmgn5* gene. The *neo* cassette, flanked by *Frt* sites, was placed downstream of exon IV. Following removal of the *neo* cassette together with exons II, III, and IV of *Hmgn5*, breeding of *Hmgn5* ^+/*tm1*^ mice gave rise to homozygous mutants lacking the nucleosomal binding domain. Black arrowheads with F, R1 and R2 show the positions of the primers used for genotyping of mice. **C)** Genotyping of female *Hmgn5^+/tm1^, Hmgn5^+/+^,* and *Hmn5^tm1/tm1^* (or male, *Hmgn5^tm1/y^)* mice. **D)** Immunofluorescence analysis of liver tissue from *Hmgn5^+/y^* and *Hmgn5^tm1/y^* littermate mice.

The nomenclature of the genetically altered mice confirms to the nomenclature recommended by the mouse genome nomenclature committee and is used by the Jackson laboratory. The superscript *^tm1^* denotes “targeted mutation #1”. The *Hmgn5* gene is located on chromosome X therefore male *Hmgn*5*^y/tm1^* do not contain an untargeted allele. The targeting vector for generating the conditional to *Hmgn5* ***^tm1/tm1^*** mice was constructed by a recombinogenic cloning strategy [19] using a murine BAC clone, RP23-145N17. The vector was constructed to remove exons II, III, and IV which code for the nucleosomal binding domain of HMGN5 (Fig. 1). A 28.8 kb fragment containing the *Hmgn5* gene was retrieved from the BAC clone into the targeting vector PL253 by recombination in the DY380 bacteria strain. The *neo* gene with the phosphoglycerate kinase 1 promoter (*pGKneo*) was employed as a positive selectable marker and the pGK-thymidine kinase cassette was used as a negative selectable marker [20]. The *loxP/Frt*-flanked positive selectable marker and the *loxP* site for conditional deletion of the HMGN5 exons were inserted as described in Figure 1. Electroporation and selection were performed using the v6.4 ES cell line as described elsewhere [20]. DNAs derived by G418/FIAU resistant ES clones were screened with a diagnostic BamH I restriction enzyme digestion using a 5′ probe external to the targeting vector sequence. Two independent targeted ES cell clones for the *Hmgn5* gene injected into C57BL/6 blastocysts generated chimeras that transmitted the mutated allele to progeny [21]. The Neo cassette was removed by crossing with *FLP*-mice, and the genomic fragment containing exons II, III, and IV of the *Hmgn5* gene was removed by crossing with *EIIA-Cre* mice. The mice containing the targeted allele were backcrossed into the C57BL/6 background for at least 5 generations. HNGN5 knockout mice were designated *Hmgn5^tm1/Y^* and their wild-type littermates denoted a *Hmgn5^+/Y^*. Mice were bred in a specific, pathogen-free facility with food and water *ad libitum*.

### Immunostaining of Liver Section

The immunofluorescence assay was performed as previously described [11]. The primary antibodies used were rabbit anti-mouse HMGN5 (2.8 µg/ml) prepared as described [14] and visualized with Alexa Fluor 488 secondary antibody (Invitrogen).

### Mouse Sample Collection

Food (NIH31 standard chow) and water was provided *ad libitum*. Standard 12-h light/dark cycles were used. Wild type and *Hmgn5^tm1/Y^* male mice were placed in metabolic cages (Tecniplast USA, Exton, PA) for 24 h to collect urine samples on three separate occasions to acclimatize mice, separated by at least 24 h in a traditional cage. At 10–12 weeks of age (n = 13), mice were killed by CO_2_ asphyxiation, and tissues were harvested and frozen in liquid N_2_. All animal studies were approved by the National Cancer Institute Animal Care and Use Committee.

### UPLC-ESI-QTOF-MS Metabolomics of Mouse Samples

Urine (1∶5 dilution) was collected and diluted with 62.5% acetonitrile containing 0.5 µM of the internal standard chlorpropamide and centrifuged at 18,000 g for 20 min at 4°C to remove precipitated protein and other particulates, and the supernatant was transferred to an autosampler vial. Liver tissue samples were homogenized in a solvent comprised of 50% acetonitrile and HPLC grade water containing 0.5 µM chlorpropamide as the internal standard. Following homogenization, liver samples were agitated on a shaking platform at 1000 rpm for 20 minutes at 30°C, centrifuged at 18,000 g for 20 min at 4°C, and the supernatant was transferred to an autosampler vial. Samples (5 µl/injection) were subjected to reverse-phase chromatography on a 50−× 2.1-mm ACQUITY 1.7 µm BEH C_18_ column (Waters Corp., Milford, MA) using an ACQUITY UPLC system (Waters Corp.) with a gradient mobile phase comprising 0.1% formic acid and acetonitrile containing 0.1% formic acid. A 0.5 ml/min flow rate was maintained in a 10-min run. The eluent was introduced directly into a Waters Q-TOF Premier mass spectrometer by electrospray ionization operating in either positive (ESI+) or negative (ESI−) ionization mode. For mass spectrometry scanning, the data were acquired in the centroid mode from 50–850 m/z. To confirm the identity of markers, authentic standards were compared with urine samples for retention time and tandem mass spectrometry fragmentation pattern when collision energies ranging from 15–35 V were applied.

### Chromatogram Deconvolution

The mass chromatographic data were aligned using MarkerLynx software (Waters) to generate a data matrix consisting of peak areas corresponding to a unique *m/z* and retention time. For urine samples, the peak area corresponding to protonated creatinine (*m/z* = 114.0671^+^, retention time = 0.31 min) was used to normalize the peak areas of other ions in a sample. This procedure minimized differences in analyte concentrations that were due to variations in renal physiology. For liver metabolomics, peak areas were normalized according to tissue weight. Data from ESI^+^ and ESI^−^ were combined to generate a data matrix suitable for downstream analysis.

### Metabolite Identification and Validation

Elemental compositions of ions were determined using the METLIN metabolite database established by the Scripps Center for Metabolomics [22], [23] and Human Metabolome Database (HMDB) established by the University of Alberta, Canada [24]. Putative ion identities were validated using tandem MS by comparison with authentic compounds.

### Data Processing and Multivariate Analysis

Centroided and integrated chromatographic mass data from 50–850 m/z were processed by MarkerLynx (Waters) to generate a multivariate data matrix. Pareto-scaled MarkerLynx matrices including information on sample identity were analyzed by PCA and OPLS using SIMCA-P+ version 12.0.1 (Umetrics, Kinnelon, NJ). P_corr_ values generated by the OPLS loadings scatter S-plot as well as scores group contribution analysis were used to determine those ions that contributed most to the separation between *Hmgn5^+/Y^* and *Hmgn5^tm1/Y^* mouse samples.

### Quantification of Urinary and Hepatic Metabolites

Metabolite concentrations were determined using an ACQUITY UPLC system coupled to a Xevo-TQ triple quadrople mass spectrometer (Waters). Chromatography was as described for UPLC-ESI-QTOF-MS analysis for all metabolites with the exception of glutathione, which was performed using a HILIC method using 50×2.1 mm ACQUITY 1.7 um BEH Amide column [25]. Serial dilution calibration curves (25–0.2 µM) were generated for each authenticated marker. Samples each from wild-type and *Hmgn5^tm1/Y^* mice were diluted (20- to 500-fold) in 50% acetonitrile containing the internal standard chlorpropamide (0.5 µM) for reversed phase chromatography and alpha-aminopimelic acid (1.0 uM) for HILIC chromatography. The mass spectrometer was operated in MRM mode, and optimal transition energies for each metabolite were monitored using the following *m/z* transitions: Betaine 118 → 59+; Creatine: 132 → 165+; Creatinine: 114 → 86+; Glutathione: 308 → 179+; Pantothenic Acid: 220 → 116+; Phenylacetylglycine: 192 → 74 −. Each urine metabolite concentration is expressed as micromoles per millimole creatinine. Liver metabolites are expressed as micromoles per milligram tissue weight.

### Gene Expression Analysis

The data for total analysis of the transcriptome of livers from wild type and mutant mice is available in the GEO database under accession number GSE39062. For additional verification of selected genes, total RNA was prepared from frozen liver using Trizol reagent (Invitrogen, Carlsbad, CA) and the RNeasy mini kit (QIAGEN, Germany) was used for RNA cleanup. cDNA was synthesized from 500 ng total RNA using iScript cDNA synthesis kit (Bio-Rad, Hercules, CA). For qPCR analysis, primers crossed exon-exon junctions, and NCBI-BLAST searches confirmed sequence specificity. Fermentas Maxima SYBR Green PCR Master Mix (ThermoFisher Scientific, Waltham, MA) was used for analysis on an Applied Biosystems (Foster City, CA) Prism 7900HT system. Relative expression calculated by the ΔΔCt method using *Gapdh* mRNA as the internal control, and statistical analyses were performed using the ΔCt values. Primer sequences for gene expression analysis are available on request.

### Nuclear Isolation and DNase I Hypersensitivity Analysis

Nuclei from liver samples were isolated as previously described [26], with minor modifications. Samples were rinsed in ice cold nuclear isolation buffer A (15 mM HEPES pH 7.5, 60 mM KCl, 15 mM NaCl, 2 mM EDTA, 0.5 mM EGTA, 0.34M sucrose, 0.15 mM 2-mercaptoethanol, 0.15 mM spermine, 0.15 mM spermidine, and a protease inhibitor cocktail (Roche). Tissues were homogenized in this buffer with 10 strokes by hand of a loose glass dounce homogenizer, followed by 10 additional strokes with a tight dounce homogenizer. This homogenate was layered onto cushions of a 1∶1 mixture of nuclear buffer A and nuclear buffer B (15 mM HEPES pH 7.5, 60 mM KCl, 15 mM NaCl, 0.1 mM EDTA, 0.1 mM EGTA, 2.1M sucrose, 0.15 mM 2-mercaptoethanol, 0.15 mM spermine, 0.5 mM spermidine, and a protease inhibitor cocktail), and centrifuged at 15,000 g for 15 min at 4°C. The supernatant was discarded and the pellet was resuspended in a nuclear storage buffer (15 mM HEPES pH 7.5, 60 mM KCl, 0.1 mM EDTA, 0.1 mM EGTA, 50% glycerol, 0.15 mM 2-mercaptoethanol, 0.15 mM spermine, 0.5 mM spermidine) and stored at 4°C until use.

For DNase I digestions, nuclei prepared as described above were washed in buffer A (15 mM Tris-HCl pH 8.0, 15 mM NaCl, 60 mM KCl, 1 mM EDTA, 0.5 mM EGTA, 0.5 mM spermidine). Samples containing 50 µg genomic DNA were then diluted in buffer A containing 6 mM CaCl_2_ digested with various amounts of DNase I (Promega, Madison, WI) for 2 min at 37°C. Digested DNA was incubated overnight with proteinase K (100 µg/mL final concentration) at room temperature, followed by extraction with water-saturated ether. Purified DNA was amplified by qPCR using the ABI Prism 7900HT system. qPCR reactions were performed using SYBR Green master kit (ThermoFisher). The primer sequences used for amplifications are available on request. Two intergenic regions known to be insensitive to DNase I digestion were used as loading controls, and the Ct of the average of both intergenic regions was used as the normalization control (ΔCt). The mean Ct of both intergenic regions was subtracted from the Ct value for each amplicon (ΔΔCt), The values displayed represent the fold difference of DNA recovered relative to the undigested sample (2^−ΔΔCt^). For statistical analysis, two way analysis of variance (ANOVA) was used to determine differences between genotypes using the ΔCt values.

## Results

### Generation Hmgn5^tm1/Y^ Mice

All HMGN protein variants, including HMGN5, interact with chromatin through a conserved region, the nucleosome binding domain (Fig. 1A). HMGN mutants that lack this region do not bind to nucleosomes and do not significantly affect chromatin structure and chromatin related activities, including transcription. In this study we aimed to minimize genomic alterations and therefore we excised from the *Hmgn5* gene, located on chromosome X, only the region corresponding to exons II, III, and IV which code for the nucleosomal binding domain of the protein [14]. The strategy for generating these mice is detailed in the methods section and summarized in Figure 1. Genomic analysis with primers F and R2 (Fig. 1B) verified complete loss of exons II-IV (Fig. 1C) and immunofluorescence analysis of liver tissues from male *Hmgn5^+/y^* and *Hmgn5^tm1/Y^* mice, reveal loss of the HMGN5 protein from the nuclei of the genetically altered mice (Fig. 1D). Analysis of cell extracts from these mice occasionally reveals the presence of a truncated protein, and immunofluorescence occasionally reveals faint signals in the cytoplasm, suggesting that the truncated gene can be transcribed.

### Liver Function in Hmgn5^tm1/Y^ Mice

Blood chemistry analysis revealed differences between *Hmgn5^tm1/Y^* and their *Hmgn5^+/y^* littermates in several parameters [10]; the most significant differences are listed in Table 1. In addition, several parameters narrowly missed statistical significance, such as fasting triglycerides (p = 0.053), both fed (p = 0.07) and fasting cholesterol (p = 0.09), and alanine aminotransferase (p = 0.08), all of which were mildly elevated in *Hmgn5^tm1/Y^* mice [10].

**Table 1:** Blood chemistry values affected by the loss of HMGN5.

Measure	*Hmgn5^+/+^* (mean ± SD)	*Hmgn5^tm1/y^* (mean ± SD)	P-value
**Overnight Fasted Mice**			
Fasting triglycerides (mmol/L)	1.24±0.3	1.58±0.4	**0.05**
Fasting non-HDL cholesterol (mmol/L)	0.57±0.1	0.66±0.1	**0.027**
**Fed Mice**			
Albumin (g/L)	25.4±0.8	26.4±1.1	**0.032**
Pancreatic α-amylase activity (U/L)	664±67	583±55	**0.01**
Calcium (mmol/L)	2.35±0.0	2.43±0.1	**0.01**

Table list altered parameters that differed significantly between *Hmgn5^+/+^* and *Hmgn5^tm1/Y^* littermate mice. 10 male mice of each genotype were analyzed.

### Metabolomic Analyses Reveals Reduced Glutathione in the Liver of Hmgn5^tm1/Y^ Mice

To determine the mechanism for the mild hepatic dysfunction suggested by the blood chemistry, metabolite changes in liver and urine samples from *Hmgn5^+/Y^* and *Hmgn5^tm1/Y^* mice, were analyzed by ultraperformance liquid chromatography coupled to electrospray ionization quadrupole time-of-flight mass spectrometer (UPLC-ESI-QTOF-MS), operating in both positive and negative ionization mode. The mass to charge (*m/z*) ratio and retention time and the abundance data generated, were subjected to principal components analysis (PCA) and orthogonal projection to latent structures data analysis (OPLS-DA).

**Figure 2:**
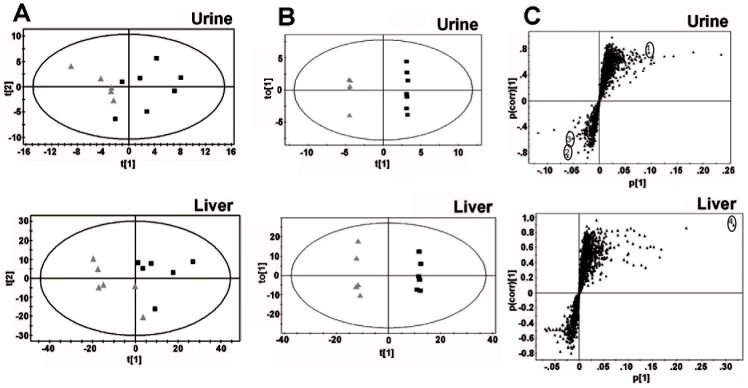
Analysis of *Hmgn5^tm1/Y^* and *Hmgn5^+/y^* littermate mouse liver and urine samples using UPLC-ESI-QTOF-MS-based metabolomics. **A.** Principal components analysis (PCA) plots of mouse liver and urine demonstrating separation between *Hmgn5^+/y^* and *Hmgn5^tm1/Y^* mice. Samples from the tissue were subjected to UPLC-ESI-QTOFMS. The PCA model with accompanying scores plot was generated using MarkerLynx data matrix. t [1] and t [2] correspond to principal components 1 and 2, respectively. Black squares indicate samples from individual *Hmgn5^tm1/Y^* mice, grey triangles indicate samples from individual *Hmgn5^+/y^* mice. Data presented were obtained in positive ionization mode (ESI+). **B.** Orthogonal projection to latent structures (OPLS) plots demonstrating separation of *Hmgn5^+/y^* and *Hmgn5^tm1/Y^* mice in urine and liver samples. Each point represents an individual mouse. **C.** S-plots showing ions important to the clustering of urine and liver samples generated from the OPLS model. Each point represents an individual ion. The p(corr) [1] P-values represent the interclass difference and p [1] P-values represent the relative abundance of the ions. All the data presented were obtained in positive mode (ESI^+^). The upper right quadrant shows ions increased in the *Hmgn5^tm1/Y^* samples, while the lower left quadrant shows the ions depleted in *Hmgn5^tm1/Y^* samples. The ions used to identify the compounds that differed between the two genotypes are listed in Table 2 and identified by the encircled numbers in panel C, are as follows: 1, Pantothenic Acid; 2, Betaine;3, Creatine;4, Glutathione.

**Figure 3:**
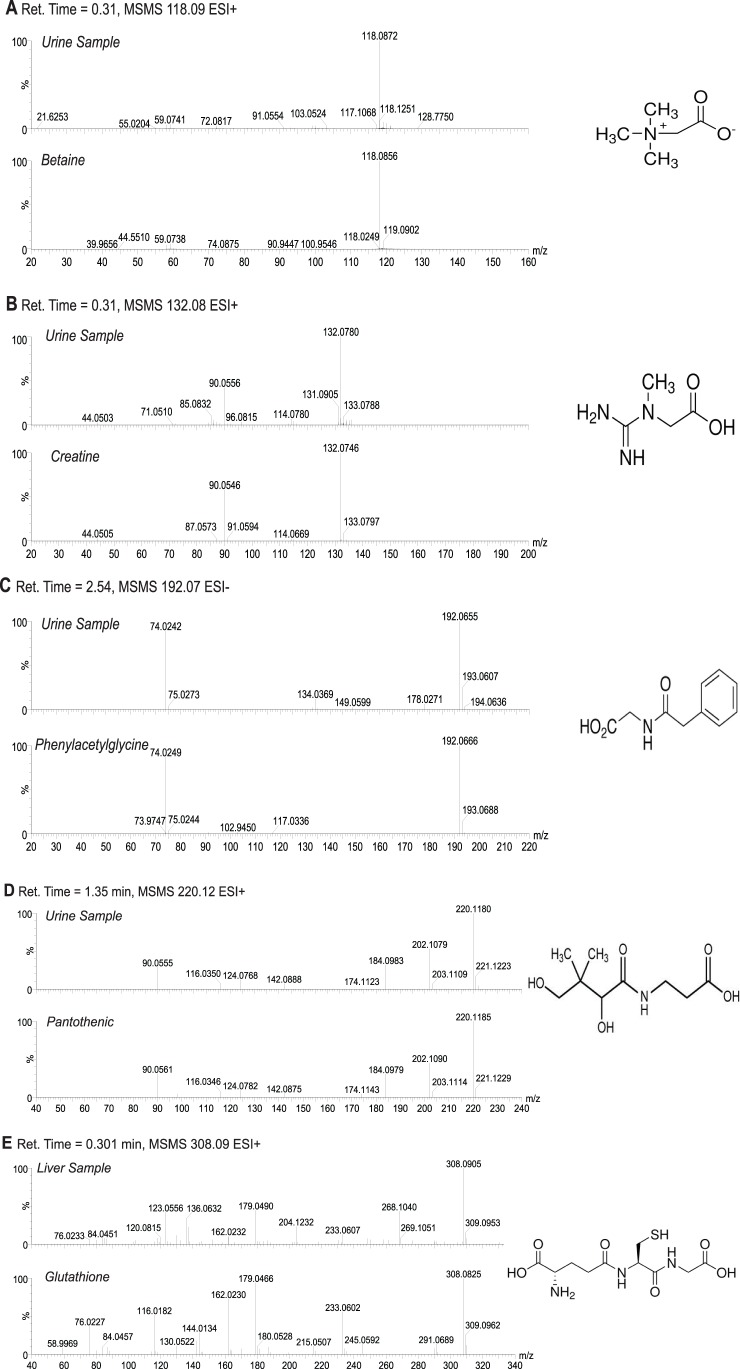
Authentication of metabolites identified in urine and liver of *Hmgn5^tm1/Y^* and *Hmgn5^+/y^* littermate wild mice. MSMS fragmentation patterns (A–E) were compared against authentic standards. All spectra were acquired in positive mode (ESI+) with the exception of phenylacetylglycine (C), which was acquired in negative mode (ESI−). Retention time and mass of the parent compound are indicated for each sample and standard comparison.

In both urine and liver, the PCA analysis distinguished *Hmgn5^tm1/y^* mice from their *Hmgn5^+/y^* littermates (Fig. 2A). Further supervised analysis by OPLS-DA resulted in even a greater degree of separation between the two genotypes, in both liver and urine (Fig. 2B). The loadings S-plot generated from OPLS that revealed the ions that gave rise to the separation between the mouse lines, identified several prominent differences in ions between genotypes in both the liver and urine (Fig. 2C). Possible structures for the ions were determined by searching the Madison Qingdao Metabolomics Consortium Database and the Scripps Center for Metabolomics and Mass Spectrometry online databases [22]–[24]. Identifiable ions with high P_corr_ values were selected for further analysis (Table 2). Many correlating ions were Na+ adducts or mass fragments of the parent compounds, and for simplicity, only identified parent compounds are shown in Table 2. Comparison of retention time and mass fragmentation pattern to authentic standards confirmed the identity of these ions (Fig. 3).

**Table 2:** Summary of identifiable ions differentially present between *Hmgn5^+/+^* and *Hmgn5^tm1/Y^* mice.

Liver							
Scores Contribution	P_corr_	Ret. Time (min)	m/z (ESI+)		Mass Error (ppm)	Empirical Formula	Identity
			Observed	Calculated			
25.20	0.893	0.3007	308.0905	308.0916	−3.570	C_10_H_17_N_3_O_6_S	Glutathione
**Urine**							
**Scores Contribution**	**P_corr_**	**Ret. Time (min)**	**m/z (ESI+)**		**Mass Error (ppm)**	**Empirical Formula**	**Identity**
			**Observed**	**Calculated**			
6.98	0.742	1.3519	220.1180	220.1185	−2.271	C_9_H_17_NO_5_	Pantothenic Acid
−5.17	−0.859	0.3078	118.0872	118.0868	3.387	C_5_H_11_NO_2_	Betaine
−2.88	−0.583	0.305	132.0780	132.0773	5.300	C_4_H_9_N_3_O_2_	Creatine
**Scores Contribution**	**P_corr_**	**Ret. Time (min)**	**m/z (ESI−)**		**Mass Error (ppm)**	**Empirical Formula**	**Identity**
			**Observed**	**Calculated**			
13.08	0.723	2.5496	192.0655	192.0661	−3.124	C_10_H_11_NO_3_	Phenylacetylglycine

Scores contribution = weighted difference between data point and average of the model; P_corr_ = modeled correlation or confidence; m/z = mass to charge ratio; ESI+ = Positive Electrospray Ionization mode; The position of the ions in an S plot generated from the OPLS is shown in Figure 2. ESI− = Negative Electrospray Ionization mode; Ret. Time = Retention Time.

The OLPS-DA analyses indicates that the most prominent change in liver resulting from functional loss of HMGN5 is the reduced form of glutathione, a compound involved the control of oxidative stress and several other metabolic processes [17]. In urine, a decrease in phenylacetylglycine, betaine, and creatine was observed, all of which are metabolites of glycine, a precursor to glutathione [17]. In addition, changes in pantothenic acid, a B-vitamin reported to have a positive effect on glutathione synthesis [27], was detected.

**Figure 4:**
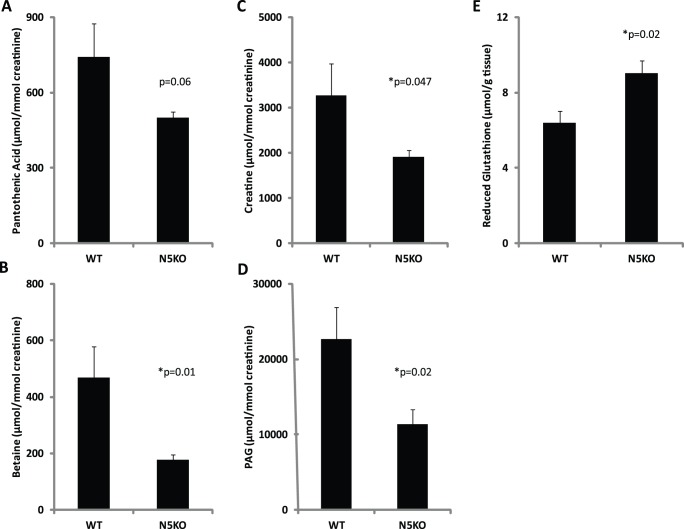
Quantification of selected ions identified by UPLC-ESI-QTOF-MS based metabolomics. Metabolite concentrations were determined by triple-quadrupole mass spectrometry and normalized to millimoles of creatinine for urine samples (A–D) and to milligrams of tissue weight for liver samples (E). P-values for differences in metabolite concentrations between genotypes are indicated (* = statistically significant, p<0.05). PAG: Phenylacetylglycine. WT: *Hmgn5^+/y^*; N5KO: *Hmgn5^tm1/Y^.* Analyses were done with 7 N5KO and 5 WT mice.

Quantification of metabolites was carried out by use of specific ion monitoring with standards and triple-quadrupole mass spectrometry (Fig. 4). *Hmgn5^tm1/Y^* mice had a 41% elevation (p = 0.018) in hepatic glutathione and a significant decrease in the urinary phenylacetylglycine (p = 0.023), a glycine conjugate. In addition, both creatine and betaine, compounds which are synthesized from glycine, were significantly reduced in the *Hmgn5^tm1/Y^* mice, by 42% and 52% respectively. Urinary pantothenic acid was also reduced compared to wild-type littermates; however, this difference narrowly missed statistical significance (p = 0.056). In summary, data from the metabolomics screening suggested a defect in glutathione utilization in *Hmgn5^tm1/Y^* mice, prompting further investigation into the molecular mechanism.

### HMGN5 Affects Gxp6 and Hk1 Gene Expression

**Figure 5:**
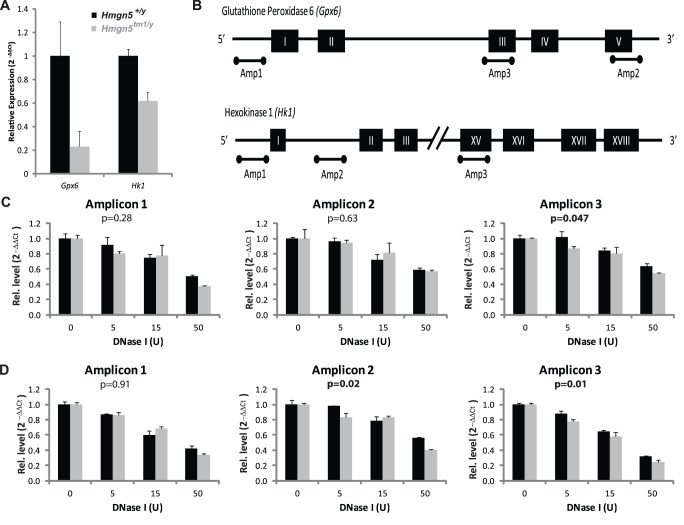
Gene expression and DNase I hypersensitivity analysis of *Gpx6* and *Hk1*. A) Relative expression of *Gpx6* and *Hk1* measured by real-time PCR. B) Maps depicting the genomic regions selected for analysis by DNase I digestion. Black rectangles indicate exon regions within the gene. Bars below the maps indicate the regions chosen for DNase I hypersensitivity analysis. (C) Recovery of *Gpx6* amplicons following digestion with varying concentrations of DNase I (D). Recovery of *Hk1* amplicons following digestion with varying concentrations of DNase I. P-values represent the significance testing for the effect of genotype as determined by two-way ANOVA, values in bold are significant at p<0.05. Analyses were done with 2 technical replicates of 3 individual mice of each genotype.

To examine the alterations in gene expression that could lead to the changes in metabolites between the *Hmgn5^+/Y^* and *Hmgn5^tm1/Y^* mice, microarray analysis was carried out [10]. Comparative analysis of the gene expression in the liver of *Hmgn5^+/Y^* and *Hmgn5^tm1/Y^* revealed that loss of HMGN5 altered the expression of 97 genes. Significantly, two of the genes that were most affected were related to glutathione metabolism: glutathione peroxidase 6 (*Gpx6*), which was the gene with was most down regulated (2^−4.08^) and hexokinase 1 (*Hk1*) which was down regulated by more than 4 fold (2^−2.32^) in the liver of *Hmgn5^tm1/Y^* mice. qPCR analysis of RNA obtained from the liver of a new cohort of mice validated the results of the array (Fig. 5A). *Gpx6* is an isoform of a glutathione-dependent enzyme responsible for the neutralization of hydrogen peroxide as well as reducing lipid hydroperoxides [17], , while *Hk1* is an isoform of a pentose phosphate shunt pathway enzyme involved in the generation of NADPH and ultimately the regeneration of reduced glutathione [17]. Thus, changes in the expression levels of these enzymes may contribute to the alterations in hepatic glutathione concentrations potentially affecting liver function.

Given that HMGN5 can alter chromatin compaction [15], we tested whether the functional loss of HMGN5 altered the DNase I sensitivity of either *Gpx6* or *Hk1* chromatin. Nuclei isolated from the liver of *Hmgn5^+/Y^ and Hmgn5^tm1/Y^* mice were digested with DNaseI and the amount of undigested DNA in 3 distinct genomic regions of *Gpx6* or *Hk1* (Fig. 5B), was quantified by qPCR using region-specific primers. The yield of the resulting amplified fragment is a measure of the relative rate of DNA digestion in chromatin. This analysis revealed that although loss of HMGN5 did not cause major alteration in the chromatin structure of the genes, it did increase the DNaseI sensitivity of amplicon 3 in *Gxp6* and amplicons 2 and 3 in *Hk1* (Fig. 5C–D), suggesting that loss of HMGN5 leads to changes in the chromatin structure of these genes.

## Discussion

This study revealed that loss of HMGN5, a nucleosome binding protein that affects chromatin structure and function, alters the metabolomic profile of liver and urine. Metabolomic analysis of both liver and urine clearly separated the wild-type *Hmgn5^+/Y^* mice from their mutant *Hmgn5^tm1/Y^* littermates, while transcriptional analysis and DNase I digestion studies link these changes the altered chromatin structure and expression of *Gpx6* and *Hk1.* Thus, the results provide insights into the biological function of this HMGN variant.

The most prominent difference between the livers of *Hmgn5^tm1/Y^* and *Hmgn5^+/Y^* mice was an elevation in hepatic glutathione concentrations, approximately 41% higher when quantified using triple-quadrupole mass spectrometry. In urine, metabolomic analysis demonstrated that *Hmgn5^tm1/Y^* mice had significantly decreased concentrations of betaine, creatine, and phenylacetylglycine, all of which are metabolites of the glutathione precursor glycine, a finding that is in agreement with previous results suggesting that glycine is a requisite for maximal glutathione synthesis [17]. The urinary metabolomics findings are consistent with the elevated hepatic glutathione concentrations, as they suggest that less glycine is available to be metabolized into these compounds, conceivable because more glycine was used for the synthesis of glutathione.

The level of hepatic glutathione is regulated by a variety of factors such as insulin [28], [29] and estrogen [30], and change during cell cycle progression and cell proliferation [31]. Because *Hmgn5^tm1/y^* mice did not show any obvious abnormalities in any of these factors we suggest that the observed changes in glutathione levels are due to the altered expression of the *Gpx6* and *Hk1* genes involved in the utilization and synthesis of glutathione, respectively. Impairments in glutathione utilization by glutathione peroxidases such as GPX6 could account for the mild elevation in the reduced form of glutathione observed. Furthermore, reduced glutathione is regenerated from its oxidized form through an NADPH dependent reaction catalyzed by the enzyme glutathione reductase [17], [18]. NADPH can become a limiting factor in this reaction, wherein additional NADPH can be generated through the pentose phosphate shunt. A key regulatory step in this pathway is the action of hexokinase, which catalyzes the penultimate reaction in the generation of NADPH [17]. Therefore, the decrease in *Hk1* expression could be a consequence of the elevation in glutathione, as sufficient glutathione production could diminish the need to generate NADPH via the pentose phosphate shunt.

Deregulation of glutathione metabolism has been implicated in several diseases including liver dysfunction [28], [30], [31], [32]. Glutathione affects hepatic metabolic processes such as detoxification and the control of oxidative stress, and therefore proper regulation of the regeneration of reduced glutathione is paramount for proper hepatic function [17]. Thus, the mild hepatic abnormalities observed in the *Hmgn5^tm1/Y^* mouse could be due to altered hepatic glutathione metabolism.

In conclusion, this study demonstrates that functional loss of HMGN5 disrupts glutathione metabolism, most likely due to chromatin changes that lead to altered expression of genes encoding two enzymes involved in glutathione utilization and synthesis, resulting in differences in the metabolism of this important thiol. Disruption of glutathione metabolism could lead to the mild hepatic differences between wild type and *Hmgn5^tm1/Y^* mice. Taken together with the previous finding that *Hmgn1^ tm1/tm1^* mice display an impaired ability to repair damaged DNA as well as elevated tumorigenesis [12], and that *Hmgn3^tm1/tm1^* mice are mildly diabetic [11] the present findings reinforces the general notion that the transcriptional changes resulting from loss of a specific HMGN variant [9], [10] could lead to specific phenotypes in mice.
